# Is deck B a disadvantageous deck in the Iowa Gambling Task?

**DOI:** 10.1186/1744-9081-3-16

**Published:** 2007-03-15

**Authors:** Ching-Hung Lin, Yao-Chu Chiu, Po-Lei Lee, Jen-Chuen Hsieh

**Affiliations:** 1 Institute of Neuroscience, School of Life Science, National Yang-Ming University, Taipei, Taiwan; 2 Laboratory of Integrated Brain Research, Department of Medical Research & Education, Taipei Veterans General Hospital, Taipei, Taiwan; 3 Department of Psychology, Soochow University, Taipei, Taiwan; 4 Department of Electrical Engineering, National Central University, Taoyuan, Taiwan; 5 Research Center for Integrative Neuroimaging and Neuroinformatics, National Health Research Institutes, Taipei, Taiwan

## Abstract

**Background:**

The Iowa gambling task is a popular test for examining monetary decision behavior under uncertainty. According to Dunn et al. review article, the difficult-to-explain phenomenon of "prominent deck B" was revealed, namely that normal decision makers prefer bad final-outcome deck B to good final-outcome decks C or D. This phenomenon was demonstrated especially clearly by Wilder et al. and Toplak et al. The "prominent deck B" phenomenon is inconsistent with the basic assumption in the IGT; however, most IGT-related studies utilized the "summation" of bad decks A and B when presenting their data, thereby avoiding the problems associated with deck B.

**Methods:**

To verify the "prominent deck B" phenomenon, this study launched a two-stage simple version IGT, namely, an AACC and BBDD version, which possesses a balanced gain-loss structure between advantageous and disadvantageous decks and facilitates monitoring of participant preferences after the first 100 trials.

**Results:**

The experimental results suggested that the "prominent deck B" phenomenon exists in the IGT. Moreover, participants cannot suppress their preference for deck B under the uncertain condition, even during the second stage of the game. Although this result is incongruent with the basic assumption in IGT, an increasing number of studies are finding similar results. The results of the AACC and BBDD versions can be congruent with the decision literatures in terms of gain-loss frequency.

**Conclusion:**

Based on the experimental findings, participants can apply the "gain-stay, loss-shift" strategy to overcome situations involving uncertainty. This investigation found that the largest loss in the IGT did not inspire decision makers to avoid choosing bad deck B.

## Background

Over the past few years, Damasio's [1] Somatic Marker Hypothesis (SMH) has become a central argument in affective neuroscience [2-7] and has also garnered increasing attention from neuroeconomists [8-10]. Bechara, Damasio, Damasio, and Anderson, [11] proposed the Iowa Gambling Task (IGT) as a means of sustaining the SMH. These studies of Iowa group claimed that the superiority of normal decision makers over patients with emotional deficits in IGT results from the intact somatic marker [1,12-16]. Damasio suggested that:

"*...the brains of the normal subjects were gradually learning to predict a bad outcome, and were signaling the relative badness of the particular deck before the actual card-turning*. "([1], p 220).

The IGT has four decks, namely, decks A, B, C, and D. Decks A and B cause participants to lose $ 250 on average during the course of ten trials; furthermore, the gains or losses made during each trial when using these decks are comparatively large. Conversely, decks C and D cause participants to win $ 250 on average over ten trials; moreover, these two decks involve comparatively small immediate gains or losses during each trial. In the gain-loss frequency dimension of the original thinking of Bechara, decks A and C both have balanced gain-loss frequency (5 gains and 5 losses); moreover, decks B and D have identical high-frequency gain and low-frequency loss (9 gains and 1 loss) (see Table 1). The Iowa gambling task contains different long-term outcomes in advantageous decks (C, D) and disadvantageous decks (A, B), and a counterbalancing of other variables. In some trials, participants experience one gain and one loss within a trial. Participants complete 100 trials blind to the game end. Bechara et al. [11] and Damasio, Tranel, and Damasio, [17] also loaded the IGT with many variables to generate an uncertain situation and prevent decision makers from using logic to reason. The following introduction for participants was adopted from the original IGT study and explains how the task simulates the uncertainty in real-life decisions:

**Table 1:** The gain-loss structure in the original IGT.

**Deck** **Card Sequence**	**A**	**B**	**C**	**D**
1	100	100	50	50
2	100	100	50	50
3	100, **-150**	100	50, **-50**	50
4	100	100	50	50
5	100, **-300**	100	50,**-50**	50
6	100	100	50	50
7	100, **-200**	100	50, **-50**	50
8	100	100	50	50
9	100, **-250**	100, **-1250**	50, **-50**	50
10	100, **-350**	100	50, **-50**	50, **-250**

**Final Outcomes**	**-250 ($)**	**-250 ($)**	**+250 ($)**	**+250 ($)**

**Gain-loss Frequency**	**5 gains** **5 losses**	**9 gains** **1 loss**	**5 gains** **5 losses**	**9 gains** **1 loss**

"...*The goal of the game is to win as much money as possible and, if you find yourself unable to win, make sure you avoid losing money as much as possible. I won't tell you for how long the game will continue. You must keep on playing until the computer stops. ... It is important to know that the colors of the cards are irrelevant in this game. The computer does not make you lose money at random. However, there is no way for you to figure out when the computer will make you lose. All I can say is that you may find yourself losing money on all of the decks, but some decks will make you lose more than others. You can win if you stay away from the worst decks*." ([18], p. 5474, 5475).

Bechara et al. [11,17] proposed that participants facing uncertainty are sensitive to long-term outcome with the assistance of somatic markers. As demonstrated by the studies of the Iowa group, decision makers obtain long-term benefits by gradually shifting their deck of choice from A and B to C and D, but this behavior is reversed for affective deficits.

Following careful review of IGT-related studies, it is worth emphasizing that some studies have compared the advantages and disadvantages of decks. Most studies only present data in an advantageous-disadvantageous format or subtract the mean numbers of choices for decks A and B from that for decks C and D [9,14,18-23]. Nevertheless, such presentation methods allow these researchers to disregard the detailed differences among the four decks. Perhaps, these methods are easier to avoid the difficult problem of "prominent deck B", which indicates that participants prefer deck B to the other three decks. However, this phenomenon is difficult to identify – most IGT-relevant studies utilized the summation of advantageous decks (C+D) or disadvantageous decks (A+B) in presenting their experimental results. Consequently, these experimental results will not contradict the basic assumption in IGT.

Conversely, Crone and van der Molen, [22,24] observed that the immediate reward directly influences participants during the IGT. That is, participants choose decks with high-frequency gains more frequently than those with low-frequency gains. They suggested that local choices of decision makers are guided by high-frequency gain and low-frequency loss, and concluded that long-term outcome ultimately dominates participant decisions.

Based on the basic IGT assumption, deck B has a disadvantageous long-term outcome that should gradually cause decision makers to avoid it owing to its relatively "large losses" (see Table 1). A growing number of studies utilized a four-deck format and showed that numerous participants prefer deck B to A [25-28]. Additionally, deck B is occasionally chosen more often than the advantageous decks C or D in the original IGT. Dunn et al. [7] conducted a meta-study of IGT studies and observed that normal participants and affective patients preferred decks B and D to decks A and C in certain studies [29-32]. Particularly, Toplak, Jain, and Tannock, [33] showed that deck B was chosen more than the other three decks not only by the patient group but also by the normal control. Notably, these five (out of over 100) studies all utilized the four-deck format to measure IGT performance. These studies thus have the opportunity to observe which were inconsistent with the expectations of Iowa group. When the "prominent deck B" phenomenon applies, shortsighted behavior can also be observed by normal decision makers under the uncertain situation.

However, to date few studies have directly observed the "prominent deck B" phenomenon and examined it empirically. This study thus attempts to determine whether the "prominent deck B" phenomenon exists in the IGT. If deck B is preferred by most participants, the basic assumption of IGT may need to be refined. Meanwhile, if most decision makers avoid deck B, there may be some confounding of the data not only of Toplak *et al*. but also of the other four studies [29-32], demonstrating that the "prominent deck B" phenomenon needs to be reconsidered.

This investigation utilizes a two-stage simplified version of the IGT [22,24] – namely: the AACC and BBDD versions of the IGT – to identify participant preferences. The simple design separates the frequent-gain decks (B, D) from frequent-loss decks (A, C), while retaining all uncertainty conditions of the original IGT.

Based on the basic assumption in the IGT [11,17], participants should be sensitive to long-term outcome in this simplified version of the IGT. Restated, decks C and D should be chosen more frequently than decks A and B in both this simplified version of the IGT and in the original IGT. Prior to the original version of IGT, this study added one further session (comprising an additional 100 trials) to identify participant sensitivity to long-term outcome in the second stage of the simple version IGT. If participants are sensitive to long-term outcome, decks A and B are chosen less often than decks C and D during stages 1 and 2 of the simple version IGT. Meanwhile, if participants are insensitive to long-term outcome they will choose decks A and B as often as (or more often than) they choose decks C and D, particularly during stage 2.

## Materials and methods

**Figure 1:**
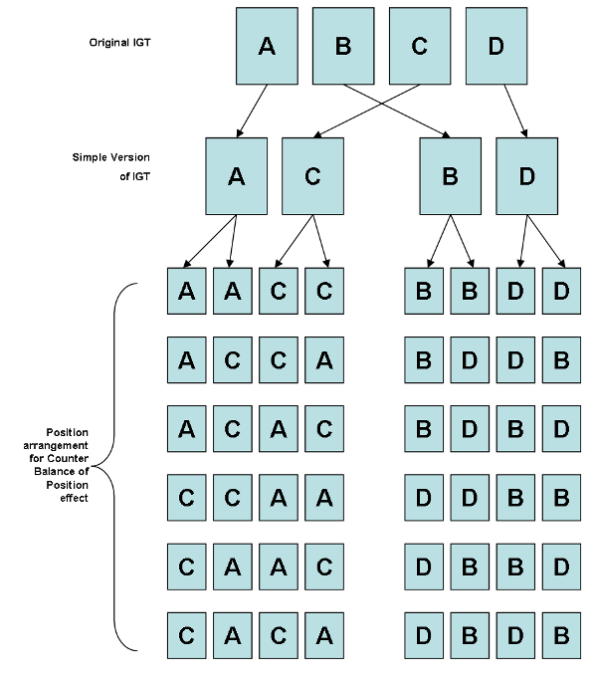
**Counterbalance of deck position in the simplified IGT**. The figure showed a flowchart regarding the generation of the AACC and BBDD versions from the original IGT and rearranged them to produce six compositions in the AACC and BBDD versions to counterbalance the position effect. Each composition was performed by two male and two female adults to control the gender effect.

This work enrolled 48 adults, mostly college and graduate students. Participants were divided into two groups: 24 participants, 12 males and 12 females, (Mean age: 22.96 years old, SD: 1.92 years) received the AACC version; and 24 participants, 12 males and 12 females, (Mean age: 23.04 years old, SD: 2.33 years) received the BBDD version. Each participant performed only one set of balanced card positions (such as, AACC, ACCA, ACAC, CCAA, CAAC, and CACA) to preclude the position effect; that is, each position arrangement (see Figure 1) was performed by four participants (2 males and 2 females). Additionally, each participant performed the game twice and their preferences during the first and second stages were traced. After completing the first stage, each participant immediately completed a questionnaire to indicate their preferences. In the two-stage design, participants were informed that they were totally free to choose the decks and that there were no time limitations in playing the game. Participants performed the second run immediately following the completion of the first session game and questionnaire. Furthermore, participants were informed that the second game was played had the same internal rules as the first game.

## Results

**Figure 2:**
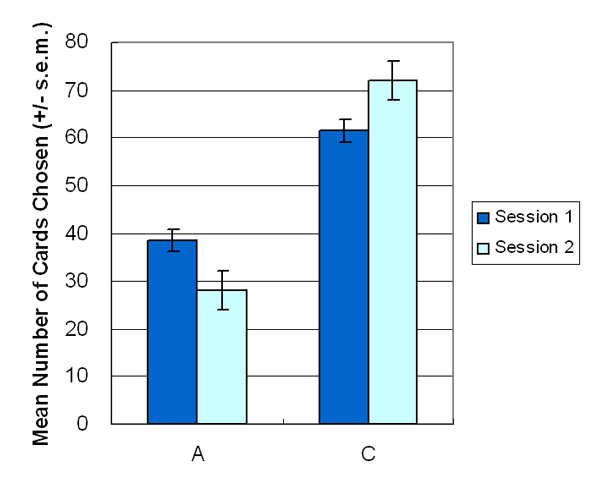
**Mean number of cards selection**. This figure illustrates the mean number of card selections between decks A and C with a summation for each stage. The mean of deck C is higher than that of deck A in both stages.

**Figure 3:**
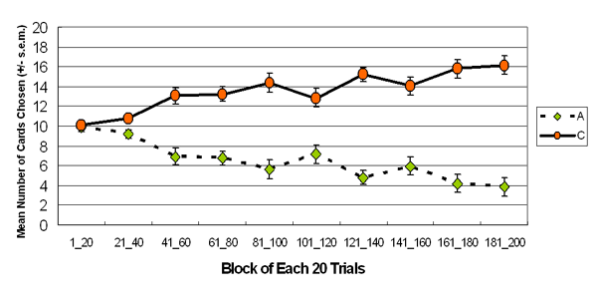
**Mean number of cards selection in blocks**. The 2-stage preference curves of decks A and C. Participants preferred deck C to deck A at the beginning and this choice pattern lasted until the end of stage 2.

The results indicated that the participants preferred deck C in both stages of AACC version (stage 1: *t *(23) = -4.76, *p *< .01 (two-tailed); stage 2: *t *(23) = -5.39, *p *< .01 (two-tailed)) (Figure 2). Furthermore, the two-factor (2 decks × 5 blocks) ANOVA (repeated measurement) was applied to test the data of each of the two stages separately. The participants shifted their preference from deck A to deck C at the beginning of the AACC version of IGT and entered the steady state before the end of stage 1. During stage 2, participants consistently selected deck C (stage 1: *F *(1, 23) = 22.68, *p *< .01; stage 2: *F *(1, 23) = 38.02, *p *< .01) (Figure 3). The descending curve of deck A and ascending curve of deck C indicated that participants can progressively shift their choice to deck C, especially during the second stage, a phenomenon that is never demonstrated by other IGT related studies.

**Figure 4:**
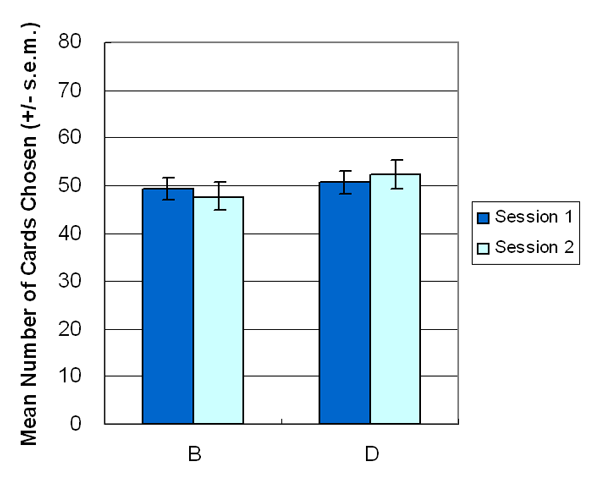
**Mean number of cards selection**. Experimental results indicated that decision makers have almost the same mean number of cards in decks B and D in stages 1 and 2. Notably, participants seemed unaware of the "largest loss" in deck B during both stages.

**Figure 5:**
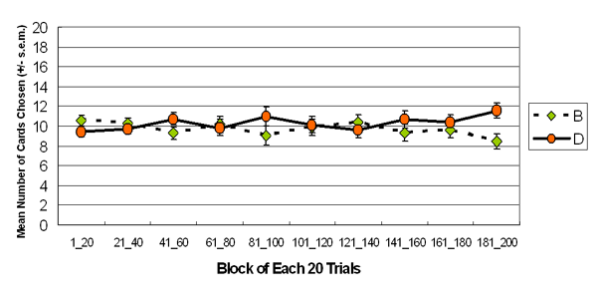
**Mean number of cards selection in blocks**. The learning curves for decks B and D in both stages indicate that participants were unaware of long-term outcome throughout stage 2. No significant differences existed between the two decks and between blocks.

**Figure 6:**
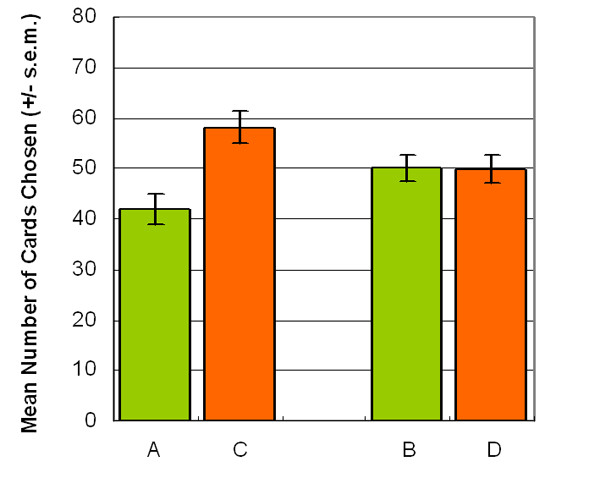
**Participant memory assessments in the simplified IGT**. Following stage 1, the two groups (AACC and BBDD) of participants were required to answer the following question: *Please recall how many trials you assigned for these decks in total 100 trials*" and most participants correctly recalled the number of cards chosen during the first stage, indicating that they have a vivid memory of each deck and that participants preferred deck C to deck A and preferred deck B as often as deck D.

In the BBDD version, participants exhibited equal preferences for decks B and D (stage 1: *t *(23) = -0.27, *p *= .78 (two-tailed); stage 2: *t *(23) = -0.79, *p *= .43 (two-tailed)) (Figure 4). The testing was performed using a two-factor (2 decks × 5 blocks) repeated measurement ANOVA. The data disclosed the inadequacy of interpreting the result of IGT based on long-term outcome. Even during the second stage, participants retained their preference for decks B and D (stage 1: *F *(1, 23) = 0.08, *p *= .78; stage 2: *F *(1, 23) = 0.64, *p *= .43) (Figure 5). The learning state curve demonstrated that participants less shifted their preference from deck B (disadvantageous deck) to deck D (advantageous deck). The two decks appear equally attractive for most participants, lasting from the beginning of the first stage through to the end of the second stage. Questionnaire data confirmed that participants preferred deck C to deck A (*t *(23) = -2.62, *p *< .05 (two-tailed)), and their preference for deck B almost matched that for D at the end of the first stage (*t *(23) = 0.06, *p *= .95 (two-tailed)) (Figure 6). In advance, the result of the final money amount which is related to final subject state of this game is also consistent with the previous observations regarding choice behavior. Most participants obtained positive final outcomes during both stages (stage 1: 20/24 participants win; stage 2: 21/24 participants win) of the AACC version. However, in the BBDD version, most decision makers obtained the final state loss not only during the first stage, but also during the second stage (stage 1: 3/24 participants win; stage 2: 7/24 participants win) (see Table 2). Mean amount of final gain-loss indicated that most subjects win money in both stages of the AACC version, while in the BBDD version, most participants lose money after stages 1 and 2.

**Table 2:** The final state of monetary gain-loss in the simplified IGT.

	AACC		BBDD	
	
Final state of gain-loss	Mean ($)	SD	Mean ($)	SD
Session 1	+539.58	722.76	-491.66	928.14
Session 2	+1319.79	1188.28	-575.00	963.91

## Discussion

The outcome of applying the AACC version is consistent with the basic assumption in the IGT regarding the long-term outcome. Following 20 trials of the game, participants began shifting their preference to deck C. According to the suggestion of Damasio, participants seemed to identify the future consequences of each deck earlier than in the original IGT. The experimental result for the AACC version is consistent with the basic assumption in the IGT.

However, contrary to the experimental results obtained by Bechara et al., the results for the BBDD version are not congruent with the basic assumption in the IGT. In the BBDD version, the intact somatic marker appears not to fully guide participants to avoid deck B (bad long-term outcome) and choose deck D (good long-term outcome). The experimental data from stage 1 confirms that decision makers are insensitive in regards to long-term outcome and may be driven by other factors. In fact, decks B and D have different long-term outcomes (Table 1), but both decks possessed the same power to guide selection behavior. According to the observation in the BBDD version, participants cannot hunch the final outcome in the long run.

To further verify the "prominent deck B" phenomenon, which implies that participants cannot make hunches regarding long-term outcomes or inability to avoid selecting the bad deck, a two-stage design was adopted for each participant. After completing both stages (200 trials), participants had experienced the IGT twice. Experimental results demonstrated that participants experienced on average "8" largest losses ($ -1250) with deck B, but did not enter the "hunch" phase of Damasio [1]. That is, even when experiencing double trials with large losses ($ -1250), the somatic marker system cannot prevent participants from choosing the disadvantageous deck B in the IGT. Obviously, the long-term outcome can interpret the result of the AACC version but not that of the BBDD version. This experimental result supports the "prominent deck B" phenomenon [7,29-33], and contradicts the basic IGT assumption.

Next, this study attempted to determine which variable can explain the "prominent deck B" phenomenon. After carefully reexamining the original structure of the IGT, this study proposes that high-frequency gain is the most plausible cause of participant preferences in the simple version IGT. In fact, if we summarized the value of "earn and pay" in each trial of IGT, the gain-loss structure between advantageous and disadvantageous decks becomes unbalanced (see Table 3); for example, the first ten-trial session of the IGT, deck A contains five gains and five losses, deck B contains nine gains and one loss, deck C contains five gains and five "standoffs", and deck D contains nine gains and one loss. Deck C obviously has better long-term outcome and gain-loss frequency than deck A. The gain-loss frequency and long-term outcome thus can be used to interpret the experimental results for the AACC. On the other hand, decks B and D possessed the same gain-loss frequency (9 gains and 1 loss) but the inverse long-term outcome. According to the Table 3, the internal structure is unbalanced for gain-loss frequency between the advantageous and disadvantageous decks. Decks B, C, and D have a relatively high-frequency gain and low-frequency loss. Thus, it is not strange that some studies show that participants prefer the disadvantageous deck B in the IGT. The present study results identified that decision makers selected decks B and D with equal frequency. Gain-loss frequency coincides closely with the results of AACC and BBDD. In contrast, long-term outcome can only interpret the results of the AACC version but not of the BBDD version.

**Table 3:** The immediate net value of each trial in the original IGT.

**Deck** **Card Sequence**	**A**	**B**	**C**	**D**
1	100	100	50	50
2	100	100	50	50
3	**-50**	100	0	50
4	100	100	50	50
5	**-200**	100	0	50
6	100	100	50	50
7	**-100**	100	0	50
8	100	100	50	50
9	**-150**	**-1150**	0	50
10	**-250**	100	0	**-200**

**Final Outcomes**	**-250 ($)**	**-250 ($)**	**+250 ($)**	**+250 ($)**

**Gain-loss Frequency**	**5 gains** **5 losses**	**9 gains** **1 loss**	**5 gains** **5 draws**	**9 gains** **1 loss**

Actually, deck B is an important index for interpreting the effect of impulse inhibition of ventromedial prefrontal function. In the proposal of Damasio and Bechara, deck B possesses a relatively large loss ($ -1250) among the four decks and negative long-term outcomes in IGT, and thus normal decision makers should be inhibited from selecting deck B owing to a small number of trials involving large losses. The basic assumption of IGT is that the largest loss can induce the robust alarm signal from the intact somatic system, guiding decision processing and inhibiting further selection of deck B. However, this study and some research groups [7,29-33] have indicated that the choice behavior of most participants is dominated by the high-frequency gain of deck B, rather than inhibited by the large loss of deck B. SMH argued that participants enter the "hunch" stage during the late period of IGT, and thus deck B was selected more frequently than the other decks. Supposing SMH is correct, a descending learning-curve should exist for deck B. However, this study demonstrated that participants seemed to prefer deck B throughout the game. Particularly, a descending learning-curve was not observed during either the first or the second sessions of 100 trials (See Figure 5).

Gain-loss frequency [34] as a powerful guiding factor in IGT and implies that decision makers can apply a "win-stay, lose-shift" strategy [34-39] when making decisions and coping with uncertain situations. Gains from the previous trial increased the probability of choosing the same deck, and immediate loss reduced the probability of remaining at the same deck [34]. Therefore, the choice pattern can be consistent with the variable, namely the gain-loss frequency. After reviewing the decision-making literature [40-43] and affective neuroscience [44-46], the above findings and some arguments of SMH also indicated that immediate gain and loss dominate choice behavior, particularly with the loading of emotion property; the results for the BBDD version thus may not be an isolated finding. Furthermore, these studies concluded that decision-makers are shortsighted, even in situations with high certainty [47].

At first glance, the difference between decks A and C results from the manipulation of long-term outcome. Careful analysis of the sum of gains and losses for each trial determined that deck A contains 5 gains and 5 losses, and deck C contains 5 gains and 5 "standoffs" (See Table 3). Decks A and C contain 5 gains, but deck C has 5 "standoffs" and thus is superior to deck A in the loss-frequency domain. Summarizing the results of two simple versions of the IGT, it can be concluded that gain-loss frequency rather than long-term outcome is the main guiding factor under uncertainty. This study identifies a divisive phenomenon of deck B inside the IGT [7,29-34,48].

IGT is the core task in constructing the SMH, suggesting that the physiological bodily feedback (Body loop) or affective brain system (As if loop), particularly the medial frontal cortex, plays a role in making long-term beneficial decisions. The SMH provided the affective brain system with a new role in making "rational" decisions, very different from other affective theories [5,44,45]. Supposing the finding of original IGT applies, this suggests that the affective system can be simulated to an internal bank which operated precisely for the long-term calculation implicitly. However, some evidence was used by the IGT to point out the instability of bodily feedback [26,49] and somatic system [25,30,50] on the physiological level. On the other hand, if the "prominent deck B" phenomenon always occurs, this implies that the influence of high-frequency monetary rewards can exceed that of high-intensity punishment under uncertainty. To summarize, the argument of SMH should be carefully reconsidered on both the physiological and task levels.

## Conclusion

Notably, IGT has been utilized for many neurological and psychiatric assessments. The present experiment utilized the simple version of the IGT to observe changes in participant preferences by separating high-frequency gain decks (B, D) from low-frequency gain decks (A, C) in two-stage games. The AACC version confirmed the result of the original finding of IGT. Nevertheless, the experimental result for the BBDD version verified the phenomenon of "prominent deck B", which is incongruent with the basic assumption of the IGT. The largest loss in deck B did not prevent participants from selecting this bad final-outcome deck. However, the high-frequency loss of deck A can prevent this choice being made. We propose that gain-loss frequency can be used to interpret the deck B phenomenon. This work concluded that these "prominent deck B" studies [7,29-34] were not an isolated finding, and the gain-loss frequency rather than long-term outcome could predict participant preferences in these similar gambling tasks. Participant preference for the high-frequency gain deck B had two implications. First, the largest loss did not inhibit normal decision makers from choosing disadvantageous deck B; second, the bad long-term outcome did not trigger participant avoidance of deck B under uncertainty.

## List of Abbreviations used

SMH: Somatic Marker Hypothesis

IGT: Iowa Gambling Task

## Competing interests

The authors certify that the information listed above is complete to the best of our original research. The authors declare that they have no competing interests.

## Authors' contributions

CH and YC have the equal contribution to this work. In detail, both have made the equal contributions to thought, design, and data interpretation as well as drafting key concept of the manuscript and refining it critically. Particularly, the original design of questionnaire was constructed by YC, namely asking participants have the distribution of 100 trials for their final memory assessment. CH carried out the data acquisition in this work. PL worked on the computerization of task, consulting of data analysis, recruiting part of subjects. JC participated in recruiting part of subjects, setting up the experimental environment. All authors gave final approval of the version to be published.
